# Collaborative care model versus usual care for people with musculoskeletal conditions and co-existing anxiety and depression: protocol for a feasibility mixed-methods randomised controlled trial

**DOI:** 10.1192/bjo.2023.80

**Published:** 2023-06-15

**Authors:** Maria Joao Cardoso Teixeira, Rokhsaneh Tehrany, Anju Jaggi, Refah Ahmed, Lucy Dove, Parashar Ramanuj

**Affiliations:** Department of Nursing, Royal National Orthopaedic Hospital NHS Trust, London, UK; Department of Therapies, Royal National Orthopaedic Hospital NHS Trust, London, UK; and Department of Allied Health Sciences, London South Bank University, London, UK; Department of Therapies, Royal National Orthopaedic Hospital NHS Trust, London, UK; and Research Department of Orthopaedics and Musculoskeletal Science, University College London, UK; Department of Therapies, Royal National Orthopaedic Hospital NHS Trust, London, UK; Nuffield Orthopaedic Centre, Oxford University Hospitals NHS Foundation Trust, Oxford, UK; and Nuffield Department of Clinical Neurosciences, University of Oxford, Oxford, UK; Department of Psychiatry, Royal National Orthopaedic Hospital NHS Trust, London, UK

**Keywords:** Anxiety disorders, collaborative care model, depressive disorders, musculoskeletal diseases, out-patient therapies

## Abstract

**Background:**

In the UK 17.8 million people have musculoskeletal pathophysiology, which becomes universal with age. Levels of discomfort and incapability correlate with symptoms of anxiety and depression. People with sufficient symptoms who seek care can benefit from collaborative diagnosis and treatment of mental and physical health organised by a case manager. This paper presents the protocol for a feasibility trial of collaborative care in an orthopaedic setting.

**Aims:**

To determine the feasibility and acceptability of providing collaborative care for patients with musculoskeletal conditions and co-existing symptoms of anxiety and depression identified on a screening tool in a physical and occupational therapy out-patient setting.

**Method:**

A two-arm parallel-group randomised controlled trial will recruit 40 adult out-patients with at least moderate anxiety and depression, who have been referred for physiotherapy and occupational therapy. Participants will be allocated on a 1:1 ratio to collaborative care or to usual care. Co-primary outcomes will be key feasibility indicators collected at baseline and at 6 months. A qualitative study will be conducted post-intervention to explore the acceptability and potential improvements to the collaborative care model.

**Results:**

This study will investigate the use of the collaborative care model for patients with musculoskeletal and co-existing moderate or severe levels of anxiety or depression.

**Conclusions:**

The results will provide important evidence to determine a future trial.

Musculoskeletal conditions are the leading cause of disability worldwide, affecting 1.71 billion people according to the World Health Organization.^1^ In the UK alone, 17.8 million people are currently diagnosed with musculoskeletal conditions,^2^ one in five adults consult their general practitioner (GP) for musculoskeletal symptoms each year^3^ and these problems are associated with approximately 30.8 million working days lost to sickness-related absence.^2^ Musculoskeletal conditions have a bidirectional relationship with aspects of mental and social health, substantially affecting quality of life, functioning and ability to engage in social roles.^3–5^

In the UK, one in six adults meets the criteria for a mental health condition.^6^ Despite the high prevalence, mental health conditions are often unrecognised in physical healthcare settings.^7^ There are numerous reasons for this, including the stigma associated with mental illness and the symptoms of the disorders themselves.^8^ Mental health conditions such as anxiety and depression that co-occur with physical health conditions are associated with health challenges for the individual and increased utilisation of healthcare services.^5^

People with co-occurring musculoskeletal conditions and symptoms such as anxiety and depression report higher pain intensity, lower quality of life and greater work absence.^3^ Depression in people with rheumatoid arthritis has been found to increase disability and reduce physical activity and concordance with treatment recommendations.^4^ In people with osteoarthritis, anxiety and depression negatively affect all domains of quality of life.^5^ Multiple studies have suggested that adequate treatment and support for anxiety and depression may therefore improve clinical outcomes for this patient population and also reduce the financial burden that these symptoms impose on healthcare services.^4,5^

A possible way to identify and improve clinical outcomes for people with both musculoskeletal problems and anxiety and depression is by implementing collaborative care.^9^ The ‘collaborative care model’ (CCM) was initially developed in the 1990s in the USA to facilitate multidisciplinary working between physicians, psychiatrists and clinical care coordinators,^9,10^ and it has since generated worldwide interest for its clinical and cost-effectiveness.^10^ Collaborative care involves care from three professionals: a physical healthcare provider (physiotherapist or occupational therapist), a mental healthcare provider (psychologist or psychiatrist) and a case manager who works closely with the patient to identify the mental and physical health support necessary.^10–12^ This model brings a more personalised approach to the care provided.^10^

Liaison psychiatry also has an important role to play in hospital settings in assessing and managing co-occurring mental disorders. However, in most cases, it operates on a ‘referral-and-triage’ (i.e. a reactive) approach. Collaborative care is a proactive approach that rests on screening and case identification. Collaborative care has been shown to enhance provision of liaison psychiatry in specialist settings such as renal care.^9^ The two approaches have been described by Curth et al,^11^ who are conducting a randomised controlled trial (RCT) to compare them.

Although collaborative care is not routine clinical practice in the UK,^10^ the National Institute for Health and Care Excellence (NICE)^12^ already recommends it for people with moderate to severe depression comorbid with cancer and diabetes.^13,14^ The CCM has never been tested in people with co-existing musculoskeletal and mental health symptoms, although evidence from a trial in people with chronic pain suggests that it may improve both disability and mental health outcomes.^15^ Before a definitive multicentre RCT can be recommended to determine the effectiveness of the intervention in a population with musculoskeletal conditions, there is a need to test the feasibility and acceptability of delivering the model within an orthopaedic setting. This paper describes the protocol for a trial in such a setting.

## Aims

The study will aim to determine the feasibility and acceptability of conducting a future multicentre trial comparing the collaborative care model (CCM) with usual care for people with musculoskeletal conditions and co-existing moderate or severe levels of anxiety or depression.

## Primary and secondary outcomes

The primary end-point will be the feasibility of the trial, which will inform a future power calculation. Therefore, co-primary outcomes are:

- participation (number of patients willing to consent)
- recruitment (number of participants who consented)
- retention rates and adherence to the intervention.

Secondary outcomes are based on recommendations for testing an intervention while gathering information on its potential for implementation in a real-world situation:^16^

- acceptability of self-reported outcome measures (completion rates, missing data)
- engagement with the CCM intervention
- to establish whether usage of additional healthcare resources can be estimated by participant self-report
- to explore qualitatively the acceptability of the CCM intervention for patients and staff, including barriers and facilitators to implementation
- to estimate the staff costs required to deliver the intervention.

## Method

This will be a mixed-methods, unmasked (unblinded), parallel-group RCT. This protocol was written with reference to the Standard Protocol Items: Recommendations for Interventional Trials (SPIRIT) checklist.^16^

### Study setting

This will be a single-centre study in the UK, based in the Therapies Department of a tertiary National Health Service (NHS) orthopaedic hospital within the Royal National Orthopaedic Hospital (RNOH) NHS.

### Eligibility criteria

Inclusion:

- patients over 18 years old, with a musculoskeletal problem and opting for a therapy out-patient appointment
- scoring ≥20 on the Patient Health Questionnaire Anxiety and Depression Scale (PHQ-ADS)^17^
- able to provide written informed consent and willing to participate
- able and willing to complete questionnaires and study assessments.

Exclusion:

- patients with a diagnosed mental health condition who are already receiving psychological treatment or are under the care of a specialist mental health service
- scoring <20 on the PHQ-ADS
- lacking the capacity to consent
- unable or unwilling to complete questionnaires and study assessments.

### Interventions

#### Collaborative care model – intervention arm

The CCM involves three professionals: a physical healthcare provider (physiotherapist or occupational therapist), a mental healthcare provider (psychologist or psychiatrist) and a case manager working closely with the participant. In this trial, the case manager will work with the participant to screen, identify, coordinate and target mental health support according to their individual needs. This will involve:

- developing a personalised care plan
- coordinating appointments with a psychologist or psychiatrist, if required
- monitoring monthly progress using validated questionnaires first administered at baseline
- adjusting support needs accordingly
- coordination of hospital appointments to reduce missed appointments and burden to the participant
- streamlining communication between physical and mental healthcare professionals via email or internal referrals
- provision of case manager care alongside routine physiotherapy or occupational therapy (usual care).

Participants will continue to be seen by a physiotherapist or occupational therapist as usual. Even if the participant is not seen again by the musculoskeletal specialist after a single visit, the case manager remains involved in the patient's care. The case manager can alert the musculoskeletal specialist to deterioration or lack of improvement in the participant's musculoskeletal condition, as well as refer participants for psychological or psychiatric support that provides more responsive input.

Participants who are randomised to the intervention group will be notified at the beginning of the trial that they will have a case manager only for the duration of the study. This will be clearly outlined during the consent process.

#### Usual care – control group

A physiotherapist, occupational therapist or both will assess the participants’ needs to help them work towards individualised goals that are important to them. These professionals will also inform, educate and empower participants to self-manage their physical capacity where possible. Following this initial assessment, participants will have a plan outlining their subsequent therapy, and exercises will be progressed as appropriate. Therapy will most often involve one-to-one sessions, but there may be instances where the participant will be involved in group classes. Physical therapy involves exercises and education; occupational therapy focuses on practical strategies to perform daily tasks. If the therapist identifies a need for additional mental health support, this will be requested via the GP or via an internal referral to the mental health services within the RNOH Trust, as per usual care.

### Outcomes

#### Feasibility outcomes

Feasibility will be assessed by collecting data on recruitment, retention and engagement in treatment. The following key feasibility indicators will be recorded:

- number of patients excluded from participation
- proportion of eligible patients who agree to participate
- retention rates following randomisation and at follow-up time points
- engagement with first and follow-up appointments
- completion of outcome data and rates of missing data.

### Secondary outcomes

#### Demographic data

Age, gender, ethnicity, marital status, highest qualification level and employment status will be collected at baseline.

#### Concomitant healthcare

Concomitant healthcare access for mental and musculoskeletal conditions that was not scheduled will be collected at 6 months from all participants (examples include GP appointments, specialist visits, accident and emergency department visits and other types of support, e.g. chiropractor or osteopath).

#### Patient-reported outcomes

All the study questionnaires have been validated and are widely used in both clinical and research contexts. Permissions have been obtained from copyright holders.

##### Anxiety and depression

We will use the 16-item Patient Health Questionnaire Anxiety and Depression Scale^17^ (PHQ-ADS) to measure the severity of anxiety and depressive symptoms. The reliability and validity of the PHQ-ADS have been established in adult populations.^17^

##### Quality of life

The three-level version of the five-item EuroQol-5 Dimension^18^ (EQ-5D-3L) is a standardised generic measure of health-related quality of life that is suitable for use in people with a wide range of health conditions and is recommended by NICE^12^ for economic evaluations in clinical trials. It can be completed by patients and may be used as a postal questionnaire or in an interview.

##### Physical health quality

The 14-item Musculoskeletal Health Questionnaire (MSK-HQ)^19^ assesses several domains: pain severity, physical function, work interference, social interference, sleep, fatigue, emotional health, physical activity, independence, understanding, confidence to self-manage and overall impact.

##### Level of pain

We will collect two measures of overall pain, the 11-point Numerical Pain Rating Scale^20^ (NPRS) and the Pain Disability Index^21^ (PDI). The PDI is designed to measure the degree to which aspects of patients’ lives are disrupted by chronic pain. The PDI rates seven categories of life activity on a scale from 0 (no disability) to 10 (activity is totally disrupted or prevented by pain).

##### Global change

The 15-item Global Rating of Change (GRoC) scale^22^ can indicate whether a person's overall health/condition is improving or worsening, as well as indicate the extent of this change.

### Participant timeline

**Fig. 1:**
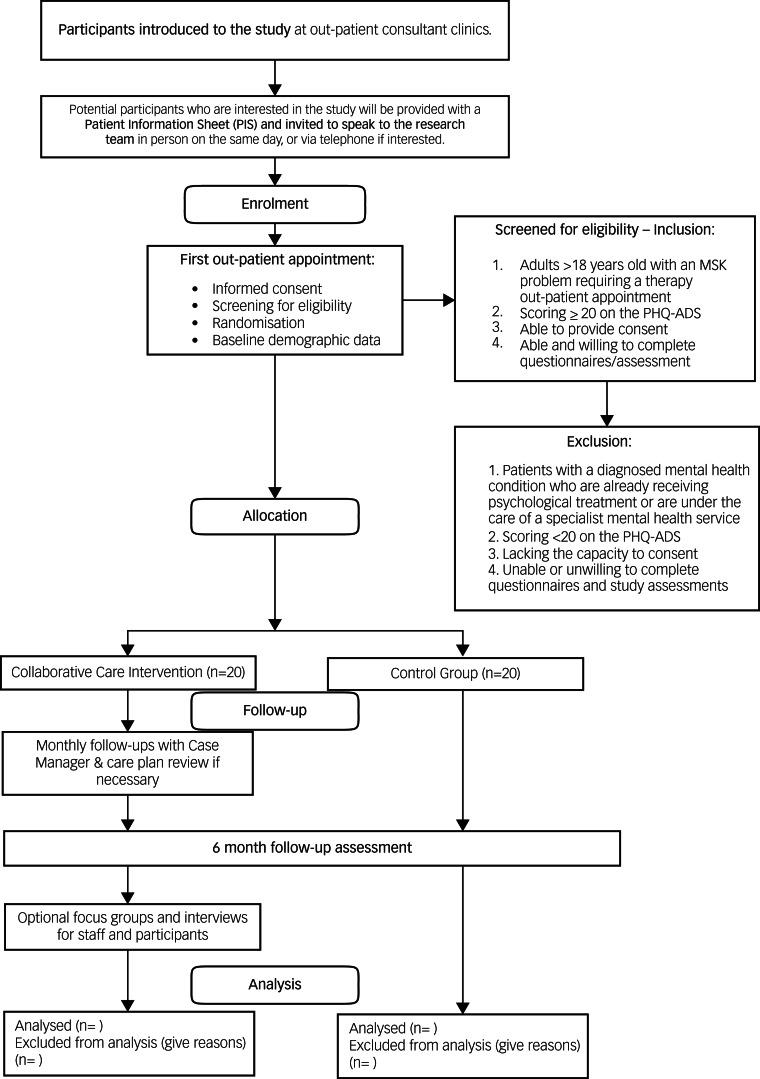
Flow diagram for the trial. MSK, musculoskeletal; PHQ-ADS, Patient Health Questionnaire Anxiety and Depression Scale.

All participants will be in the trial for 6 months. Fig. 1 shows the recruitment and flow of participants through the study.

### Sample size

As this study is a feasibility trial, all analyses will be descriptive, with no hypothesis testing. Consequently, no formal sample size calculation has been conducted. We aim to recruit 40 patients in total, with 20 randomised to each arm.

The study aims to recruit these patients over 3 months, with a target recruitment rate of 20% of all eligible patients. It is estimated that there may be at least 200 new eligible patients in 3 months. Assuming this number of eligible patients, the recruitment rate could be estimated within ±6% with a 95% confidence level.

### Recruitment

Posters will be placed in the therapies out-patient department to increase awareness of the study, and flyers will be sent out with first appointment letters. Healthcare professionals will be briefed on the participant eligibility criteria and will introduce the study to potential participants during their first appointments. If potential participants express an interest during this appointment, they will then be introduced to a member of the research team (after their appointment). The researcher will provide verbal and written information about the trial on the same day (Fig. 1).

All interested patients will be given a minimum of 24 h to consider their participation in the study, to allow them time to read the trial literature, formulate questions and discuss their participation with relevant others. A member of the research team will call or email these potential participants. Interested patients who decline participation in the study at this stage will be asked to briefly provide the reasons for their decision; however, they will not be obliged to provide a reason if they do not wish to. Permission will also be sought to obtain demographic data such as age, ethnicity and gender from any patients who are approached but decline participation. This information will be used to characterise the sample and will inform recruitment strategies for a future multicentre trial. Participants will be free to withdraw at any point during the study without needing to provide a reason. Data will be retained for all participants up to the date of withdrawal.

### Screening and enrolment

Written informed consent will be obtained from all patients who agree to participate in the trial. This will be obtained during routine musculoskeletal appointments within 3 weeks after first being approached. After obtaining informed consent the research team will complete a screening process with the participant to ensure they meet the study inclusion and exclusion criteria. This will include asking them to fill in the PHQ-ADS questionnaire. If excluded from the trial, the participant will be informed of the reason.

### Randomisation and allocation

The study will follow the Consolidated Standards of Reporting Trials (CONSORT) guidelines^23^ for the design and implementation of randomised controlled trials. The CONSORT extension for randomised pilot and feasibility trials is meant to provide reporting guidance for any randomised study in which a future definitive RCT, or part of it, is conducted on a smaller scale. After the consent and eligibility procedure, participants will be randomised in a 1:1 ratio (collaborative care:usual care). Allocations will be concealed and undertaken using online randomisation software^24^ by a member of the research team. Since this study aims to assess the feasibility of the CCM, which includes support from a case manager, it will not be possible to mask healthcare professionals or participants to the intervention.

### Data collection methods

#### Baseline assessments

After randomisation, participants from the intervention and control arms will be asked to complete baseline questionnaires, lasting approximately 60 min. These will include tailored questionnaires to collect demographic data (age, gender, ethnicity, marital status, highest qualification level and employment status), medical history and current medication usage. All participants will also be asked to complete other patient-reported outcome measures at baseline, including self-reported measures of depression, anxiety, pain and quality of life (see the ‘Patient-reported outcomes’ subsection above).

#### Follow-up assessments

Participants from the intervention and control arms will be invited to complete the same patient-reported outcome measures at follow-up assessments that were completed at baseline, i.e. self-reported measures of depression, anxiety, pain and quality of life (further detailed below). They will also be asked to provide details about any changes to their medication usage. The use of additional healthcare resources will only be recorded at 6 months. All data will be collected through questionnaires administered during face-to-face appointments and telephone or video calls, depending on participant preference and availability.

#### Embedded qualitative study: acceptability of the intervention and facilitators and barriers to implementing the CCM

Focus groups and interviews will be used to explore the views, thoughts and experiences of both participants and healthcare professionals from the intervention group. This will include exploring opinions on working with a case manager, potential advantages of the CCM, and challenges to delivering and receiving the intervention.

##### Interviews

Participants allocated to the intervention group will have the option of taking part in an interview within a month of completing the 6-month follow-up. This interview will last for approximately 60 min, with some guided topics delivered by a member of the research team. Participants can decline to take part at any point. All interviews will be planned to take place in person at the hospital. However, in the event of any COVID-19 or similar restrictions or participant preference, there will also be the opportunity to participate in interviews using a virtual platform or via telephone. Interviews will avoid breaches of confidential material regarding participants’ mental health conditions.^25^ All interviews will be audio-recorded and consent will be obtained before recording.

##### Focus group

All healthcare professionals (physiotherapists, occupational therapists, psychologists and psychiatrists) who were involved in the care of patients allocated to the intervention arm will be invited to take part in a focus group within 4 weeks of the end of the trial. We aim to invite 6–10 healthcare professionals for the focus group,^26^ which will last approximately 60 min. Focus groups will take place at the hospital and several potential dates will be offered so those invited will have the opportunity to attend the one most convenient to them. Although our preference will be to conduct focus groups in person, they might also be conducted virtually through an online platform in light of any COVID-19 or similar restrictions. We do not expect staff to attend more than one session, but they would be welcome to attend another session if they have additional feedback to contribute.

#### Usage of additional healthcare resources

The usage of additional healthcare resources will be collected through all participants’ self-reported questionnaires at the 6-month follow-up.

#### Staff costs and main resources to implement the CCM

The staff costs and main resources used for the intervention arm will be estimated from the number, type and duration of appointments performed by the case manager, therapists and mental health specialist. Data will be collected using the therapies appointment booking system.

### Data management

Participants will be allocated an anonymised unique identifier. Only the research team will be able to identify participants, using a key that links the unique identifier to an identifiable data field. Personal identifiers (i.e. the informed consent forms) will be stored separately from the main research data in a different cabinet. All electronic data relating to the study will be stored in an encrypted format, in a study-specific database accessible only to delegated members of the research team via a unique username and password.

### Data analysis

Any data collected during this study will available on request from the corresponding author (M.J.C.T.). The data will not be made publicly available, in accordance with the General Data Protection Regulation (GDPR).

#### Quantitative data

Owing to the exploratory nature of this feasibility study, the sample size will be based on the pragmatic limits of recruitment. Since the primary aim of this study is to determine the feasibility of conducting a future RCT, a descriptive analysis will be performed to examine key feasibility and process-related outcomes. All quantitative data will be uploaded into SPSS^27^ for analysis, where recruitment, retention and follow-up rates at study completion will be quantified using absolute and relative frequencies and percentages of the overall population.

In particular, the percentage of potential participants who agree to undergo the screening assessment out of the total number of potential participants who were initially invited will be calculated. The percentage of the total number of participants who were enrolled into the study out of the total number invited will be calculated with a 95% confidence interval ±6%.

The main analysis will estimate the percentage of potentially eligible participants, consent and overall uptake. The retention, follow-up and outcomes rates will be calculated in relation to the number recruited. Engagement with the intervention will be described by the percentage of appointments attended as a proportion of booked appointments. An estimated engagement rate of approximately 90% (32/36 retained patients) will give a 95% confidence interval width of ±10%. Additional healthcare resources will be described by type and frequency of use in absolute numbers.

The means and confidence intervals of clinical outcomes will be calculated to determine their sensitivity to change. This will guide the identification of a primary outcome measure for a future RCT. In addition, we will determine the acceptability of the clinical outcomes by calculating percentage rates of completion for the PHQ-ADS, EQ-5D-3L, NPRS, PDI, MSK-HQ and the GRoC.

A detailed statistical analysis plan will be developed by the study statistician. To illustrate participant flow, we will report results using a CONSORT flow diagram.^23^

#### Qualitative data

Focus group and interviews will be transcribed in full (verbatim) by an external company, checked for accuracy by a member of the research team and then imported into NVIVO version 12 qualitative data analysis software,^28^ to aid management and indexing of data. Transcripts from the participants and healthcare professionals will be analysed separately. A subset of transcripts will be independently double coded by a member of the research team and compared. Discrepancies will be discussed with another member of the research team and resolved to achieve a coding consensus. The analysis will begin shortly after data collection starts. It will be carried out by one member of the research team using normalisation process theory (NPT).^29^ This type of analysis explains the processes by which complex interventions become routinely embedded in healthcare practice.^29^

This model is particularly important for addressing the execution and realisation of interventions in a pre-existing operational context such as a healthcare setting, where novel interventions must fit into deeply embedded professional and organisational systems. NPT will be used to develop the topic guides for interviews and focus groups and provide the framework for analysis. NPT is explicitly concerned with the workability and sustainability of complex interventions. NPT can therefore be used to address the feasibility of implementation, by recognising which components of implementation may be particular barriers or facilitators by examining the extent to which the intervention can become integrated into everyday practice.^29^ These data will support a deeper understanding of the acceptability of the intervention, and the resources needed, and disclose potential barriers and facilitators from the participants’ and healthcare professionals’ points of view.

#### Cost analysis

The objective of the cost analysis will be to assess the relative cost of the clinical staff from the number, type and duration of appointments performed by the case manager, therapists and mental health specialist. Costs will be calculated using the National Cost Index^30^ for the NHS.

### Monitoring

#### Trial steering committee

Independent supervision of the trial will be carried out by members of the Trial Steering Committee (TSC). The TSC will have responsibility for monitoring the progress of the trial, engagement with the protocol, patient safety and consideration of new information. The TSC will include the chief investigator, principal investigators and three independent chairs (patients). The trial statistician will attend when appropriate. The chief investigator will oversee the overall management of the trial. The principal investigator (M.J.C.T.) will be responsible for the coordination of the study on site and will carry out the day-to-day activities involved in running the trial at each site. Delivery of collaborative care will be carried out by a designated skilled case manager (R.A.).

#### Suicidal ideation and risk of self-harm protocol

Suicidal ideation might be identified by the PHQ-ADS, which asks specifically about thoughts of self-harm. Participants might also disclose this ideation at any point during the study, from recruitment to discharge.

At present, patients may have these thoughts but are not assessed or asked about them. There is strong evidence to suggest that asking patients about suicidal thoughts does not increase risk but may be protective.^31^ Should participants disclose suicidality, the protocol outlined below will be followed.

For any participant scoring 1 or higher on question 9 of the PHQ-ADS (‘Thoughts that you would be better off dead or of hurting yourself in some way’), the research team will discuss these thoughts with the patient and ask them to answer the Columbia-Suicide Severity Rating Scale Screen Version (C-SSRS Screen). The C-SSRS Screen^32^ is a validated 6-item assessment scale for people with suicidal ideation. It categorises patients into low, medium and high risk. For example:

- passive thoughts such as wishing to be dead with no further risk indicators are considered low risk
- methods and plans, or active thoughts such as wishing to cause self-harm, are considered a moderate risk
- suicidal intent and any suicidal behaviour in the past 3 months indicate a high risk.

All triggers of the suicidal ideation and risk of self-harm protocol and the actions that are taken in response will be recorded on the Research Risk of Self-Harm form and clinical notes.

#### Suicidal thoughts before randomisation, during usual care or at the end of study interviews

The research team will inform the named clinician responsible for the participant's care of their level of risk. It is standard practice within the RNOH Trust that all thoughts of self-harm should be discussed with the RNOH Psychiatry Service. The RNOH Psychiatry Service will assess the participant and, depending on the level of risk, offer advice or refer for treatment and inform their GP. Participants will also be signposted to the Rethink Mental Illness online resources website, which provides information on coping with suicidal thoughts.

For participants who reveal suicidal thoughts for the first time during the end-of-study interview and are no longer under the care of the RNOH out-patient services, or for participants who refuse to be referred to the RNOH Psychiatry Service, the research member will discuss the participant's presentation with the chief investigator, who is a consultant psychiatrist, within 24 h.

#### Suicidal thoughts reported by participants in the intervention group

If suicidal thoughts emerge in a participant allocated to the intervention group, the case manager will assess the risk clinically, supported by the C-SSRS Screen tool. The research team will then ask for the participant's consent to make a referral according to the risk level:

- participants deemed to be at low risk will be flagged up to their GP
- participants at moderate risk will be offered triage and risk assessment by the hospital psychiatrist within 1 week of referral
- participants at high risk will be assessed immediately by the hospital psychiatrist.

**Fig. 2:**
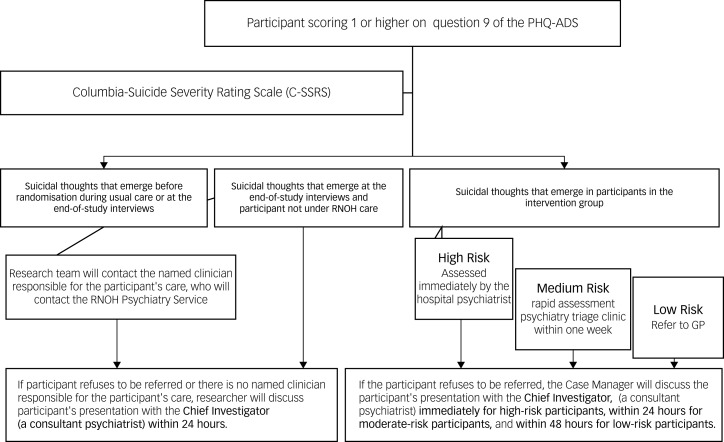
Suicidal ideation and risk of self-harm flow diagram. PHQ-ADS, Patient Health Questionnaire Anxiety and Depression Scale; RNOH, Royal National Orthopaedic Hospital; GP, general practitioner.

If a participant at moderate or high risk of self-harm refuses to be referred, the case manager will discuss the participant's presentation with the chief investigator. This will be immediately for high-risk participants, within 24 h for moderate-risk participants and within 48 h for low-risk participants (Fig. 2).

#### Patient and public involvement

Patient and public^33^ involvement was coordinated early on during the design and planning phase. In particular, patients were invited to provide feedback on the proposed length of the study and comment on the perceived burden of study participation. The patient burden was kept to a minimum following feedback. Outcome measures were carefully selected to be suited to, and well-used in, this population. Careful consideration was made to ensure that the patient information sheet provided a clear description of the CCM.

Three patients have been involved throughout the feasibility RCT development process, including involvement to refine the patient information sheet and informed consent form. Feedback on earlier versions of the patient information sheet improved the clarity of how the CCM was described. Three other patients have agreed to join the steering committee as co-applicants and attend regular TSC meetings to oversee the study.

### Ethical approval

The authors assert that all procedures contributing to this work comply with the ethical standards of the relevant national and institutional committees on human experimentation and with the Helsinki Declaration of 1975, as revised in 2008. All procedures involving human subjects/patients were approved by the East of England – Cambridgeshire and Hertfordshire Research Ethics Committee (approval number 21/EE/0257). No patients will be offered financial incentives to take part. Professionals invited to take part in a focus group will not be reimbursed for their time.

On enrolment, all participants will be de-identified and given a unique study identifier, to ensure that patients will not be identified from any data collected as part of the research project. Study numbers will be used on all documentation. To safeguard participants’ rights, the minimum personally identifiable information will be used if possible. Clinical care will continue during and after this study. Participants will not be discharged unless it is safe to do so and may be transferred to community specialist teams if required.

### Dissemination

The project itself will be presented in the monthly clinical meetings across the Trust and disseminated through the internal and public websites. Furthermore, professional associations will participate in the dissemination process through their websites, conferences and publications. At least two papers presenting results will be published in indexed peer-reviewed journals using an open access format. Authorship eligibility will follow the International Committee of Medical Journal Editors’ guidance. Results will also be presented at several national and international conferences, as well as at postgraduate research forums, at universities or at other relevant events.

## Discussion

It is still unclear how integrated care models can be disseminated and pragmatically implemented in routine care in settings with significant barriers to change (such as limited resources, professional resistance and competing priorities). These barriers are likely to be exacerbated in the current times of economic uncertainty. Trialling new strategies to reduce the burden of mental health conditions such as anxiety and depression in people with musculoskeletal problems in the orthopaedic setting could potentially improve clinical and patient-centred outcomes and reduce secondary complications and the need to access additional/ongoing healthcare services. To our knowledge, this will be the first RCT to investigate the feasibility of delivering a CCM in the context of an NHS orthopaedic out-patient setting. The findings from this trial will guide the design of a future definitive multi-centre RCT of collaborative care versus usual care for the management of patients with musculoskeletal problems and coexisting mental health conditions.

A potential limitation of this feasibility trial relates to the single-site design, as the participants from both the intervention and control arms may potentially receive care from the same treating clinician. Although the primary objective of this initial trial will be to assess key feasibility and process-related outcomes rather than conducting hypothesis testing *per se,* a future definitive trial with greater available resources should consider cluster randomisation using multiple sites to avoid contamination.

In summary, the rising prevalence of musculoskeletal problems and anxiety/depression necessitates the need to develop and implement new strategies to optimise the management of patients who present with both conditions, as these individuals are at greater risk of poor clinical outcomes and lower satisfaction and place greater pressure on NHS resources.^4,8,15^ The CCM could offer a potential solution to this problem, but the intervention has not yet been evaluated in people with coexisting musculoskeletal and moderate and severe levels of anxiety and depression in an NHS orthopaedic rehabilitation setting. Furthermore, there is a need to identify whether any pragmatic refinements are required prior to implementation and in parallel to determine whether it is feasible to conduct a future trial.

## Data Availability

The data that support the findings of this study will be available from the corresponding author, M.J.C.T., on reasonable request.
